# Amyloidogenesis: What Do We Know So Far?

**DOI:** 10.3390/ijms232213970

**Published:** 2022-11-12

**Authors:** Zeina Alraawi, Nayan Banerjee, Srujana Mohanty, Thallapuranam Krishnaswamy Suresh Kumar

**Affiliations:** 1 Department of Chemistry and Biochemistry, Fulbright College of Art and Science, University of Arkansas, Fayetteville, AR 72701, USA; 2 School of Chemical Sciences, Indian Association for the Cultivation of Science, 2A & 2B Raja S. C. Mullick Road, Jadavpur, Kolkata 700032, India; 3 Department of Chemical Sciences, Indian Institute of Science Education and Research, Kolkata 741246, India

**Keywords:** amyloids, amyloid precursor protein, amyloid structure analysis, protein aggregation, fibril formation, Aβ peptide, amyloid related diseases, physical techniques in amyloid analysis, COVID-19 and amyloidosis

## Abstract

The study of protein aggregation, and amyloidosis in particular, has gained considerable interest in recent times. Several neurodegenerative diseases, such as Alzheimer’s (AD) and Parkinson’s (PD) show a characteristic buildup of proteinaceous aggregates in several organs, especially the brain. Despite the enormous upsurge in research articles in this arena, it would not be incorrect to say that we still lack a crystal-clear idea surrounding these notorious aggregates. In this review, we attempt to present a holistic picture on protein aggregation and amyloids in particular. Using a chronological order of discoveries, we present the case of amyloids right from the onset of their discovery, various biophysical techniques, including analysis of the structure, the mechanisms and kinetics of the formation of amyloids. We have discussed important questions on whether aggregation and amyloidosis are restricted to a subset of specific proteins or more broadly influenced by the biophysiochemical and cellular environment. The therapeutic strategies and the significant failure rate of drugs in clinical trials pertaining to these neurodegenerative diseases have been also discussed at length. At a time when the COVID-19 pandemic has hit the globe hard, the review also discusses the plausibility of the far-reaching consequences posed by the virus, such as triggering early onset of amyloidosis. Finally, the application(s) of amyloids as useful biomaterials has also been discussed briefly in this review.

## 1. Introduction

It is impressive how nature accurately implements molecular self-assembly through ordered growth of nanoscale building blocks with high efficiency in order to fabricate macromolecular architectures. Peptides folding onto proteins, nucleotide chains twisting around to form the DNA duplex and cells proliferating into tissues, to list a few, are some of the most astounding biological self-assembly processes nature has to offer [1,2,3,4,5]. 

During molecular self-assembly, molecules associate under equilibrium conditions to form structurally organized frameworks, stabilized via various intermolecular interactions, such as electrostatic interactions, van der Waals forces, stacking interactions, hydrogen bonds, and hydrophobic effects. All of these, in addition to the covalent and other intramolecular interactions, produce a well-ordered supramolecular structure. The topology of monomeric units play an important role in the architecture, design, composition, and characteristics of the self-assemblies produced [6,7]. Molecular self-assembly lies at an interdisciplinary interface of protein science, biochemistry, molecular engineering, polymer science, and materials science [8]. Molecular self-assembly of biomolecules, such as peptides and proteins, has emerged as a topic of crucial interest in recent times because of its relevance in biotechnology and health sciences. Protein structures gain their functionality through complex molecular self-organization processes that lead to protein folding and are affected by nature of solvent, osmolarity, pH, temperature, etc. This makes protein folding highly sensitive to its biophysiochemical environment, often leading to protein misfoldings which are at the root of various neurodegenerative disorders [9,10,11,12]. Moreover, the interaction of a ligand with its receptor has been related to self-assembly. The morphology of molecular assemblies produced by enzyme instructed assembly of small molecules is crucially dependent on ligand–receptor interactions. For example, it has been indicated that the ligand–receptor interaction changes the morphology of molecular assemblies produced by enzyme instructed assembly of small molecules to short nanofibers. In contrast, the absence of ligand–receptor interaction directs enzymatic dephosphorylation of precursor to generate the hydrogelator that self-assembles to form long nanofibers, thus preventing the nanofiber formation [13]. 

Aromatic amino acids, such as tryptophan, phenylalanine, and tyrosine, have reportedly been shown to have a tendency towards forming fibril-like self-assemblies [14,15]. Understanding the interactions that induce and stabilize the molecular self-assembly process have become a target in structural biochemistry for reducing the risk of fibrillation of various proteins and peptides associated with different diseases and for the safe storage of various proteinaceous concoctions of clinical relevance. 

## 2. Single Amino Acid Self-Assemblies

Single amino acid self-assemblies have garnered a great deal of attention recently because of their role in metabolic disorders [16]. Single amino acids, such as phenylalanine, tyrosine, tryptophan, cysteine, and methionine have been found to cause disorders, such as phenylketonuria (PKU), tyrosinemia, hypertryptophanemia, cystinuria and hypermethioninemia, respectively [16,17,18]. Phenylalanine self-assemblies show similar properties to that of amyloid assemblies as they have characteristic birefringence, and Thioflavin T (ThT) fluorescence and electron diffraction patterns [19,20,21]. Besides being ordered like amyloid fibrils, they exhibit strong cytotoxic activities as well. Phenylalanine aggregates were observed in PKU, thus strongly indicating similar etiology of PKU with amyloid-related diseases [22,23,24]. Aromatic amino acids, such as phenylalanine, tyrosine, and tryptophan, are characterized by their high hydrophobic nature and their tendency to form β-sheet structures [19,20]. Their aromaticity has been proposed to promote amyloid fibrillation because of π-stacking that can energetically drive the self-assembly process forward and provide specific directionality and orientation through stacking [18,23]. In addition to their high hydrophobicity, low chain flexibility and β-sheet propensity, amyloid residues also have a planar steric profile that can be easily fit into ‘steric zipper’ structures in laminated β-sheets [25]. Although aromatic interactions are not necessary for amyloid formation, their presence has exhibited acceleration in the amyloid formation process [17,26]. However, not only aromatic amino acids, but also non-aromatic amino acids, such as cysteine and methionine, have been shown to self-assemble, forming very long fiber-like aggregates [27]. Inhibitors containing aromatic groups have also been synthesized. These mechanisms indicate that either assembly is blocked, or solubility of the amyloid species increases due to the introduction of an inhibitor based on the underlying principle that amyloid formation can be inhibited using modified molecular recognition elements [28]. Resveratrol is one such compound that has exhibited neuroprotective activities, such as promoting clearance of Aβ aggregations and reduction of plaques. It has been reported that resveratrol inhibits amyloid formation by IAPP and amyloid-β peptide [29]. 

## 3. The History of Amyloid Formation

In 1639, Nicholas Fontanus observed an abscess in the liver and white stones in the spleen in the autopsy of a man. This might have been the first case of sago spleen amyloidosis, a form of amyloid deposition in the spleen and the discovery of amyloids [30,31]. 

**Figure 1:**
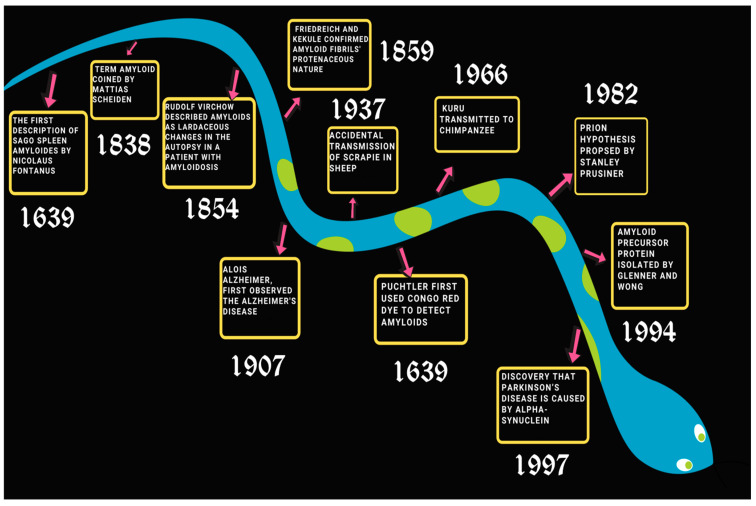
Chronology of events in the history of amyloid research.

Amyloid comes from the Latin word amylym which means starch. The term amyloid was coined by Matthias Schleiden in 1838. It was during his iodine sulfuric acid test on plants that he referred to amyloids as starch-like [30]. In medical literature, the term amyloids was used by Virchow, describing corpora amylacea found in the liver of a patient during the autopsy, as starch-like deposits since they responded to the iodine sulfuric acid test just like starch [30]. However, in 1859, Kekule and Friedrich used protein staining dyes to show that these deposits are in fact proteinaceous in nature. Further studies concluded that amyloids are protein deposits of insoluble fibrils that cause amyloidosis [32]. Prions, another set of macromolecules of contemporary interest, are closely related to amyloids. The earliest case of prion disease in humans is suspectedly reported to be *Kuru*, a brain disease found in some cannibalistic tribes of New Guinea. However, the very first time that the infectious nature of prion disease was observed was during the accidental transmission of scrapie in sheep in 1937. The protein-only hypothesis or the prion hypothesis was proposed by Stanley Prusiner in 1982, for which he later received the Nobel Prize in Medicine in 1997. Prions are infectious agents in the form of misfolded proteins, which propagate by inducing protein misfolding. Prions have been reported to cause several proteins to aggregate into tightly packed beta-sheets, called an amyloid fold, relating them to amyloids [33,34]. One of the most common neurodegenerative diseases of recent times, Alzheimer’s disease, was first observed by Alois Alzheimer in 1907. While James Parkinson had first medically described another neurodegenerative dysfunction, Parkinson’s disease, in 1817, it was only in 1997 that it was found that alpha-synuclein protein misfolding causes Parkinson’s disease [35,36]. A protein derived from the twisted β-plated sheet fibrils found in cerebrovascular amyloidosis associated with Alzheimer’s was first isolated by Glenner and Wong in 1984 [30]. In 1875 three scientists, Cornil, Heschl, and Jürgens, independently established the importance of methyl violet stain to detect amyloids [16]. Congo red (CR), an aniline dye, was first developed by German chemist Paul Böttiger [37]. Eventually, Congo red (CR) became the most predominantly used dye to detect amyloids. When stained with Congo red (CR), amyloids give an apple-green birefringence under polarized light caused by the increased optical anisotropy after binding with the dye [38,39]. However, the evidence of the CR dye showing non-specific binding and also binding with non-amyloid substances suggests using it with discretion to determine the presence of amyloids. Many other staining methods have eventually been developed, such as staining with Thioflavin T (ThT), Congo Corinth, Benzopurpurin 4B, and Acid Fuchsin to detect the presence of amyloids. However, in current times, with the advent of spectroscopic prowess, several biophysical techniques have become the primary method for the detection of amyloids (Figure 1) [37].

## 4. The Structural Features of Amyloid Fibrils

**Figure 2:**
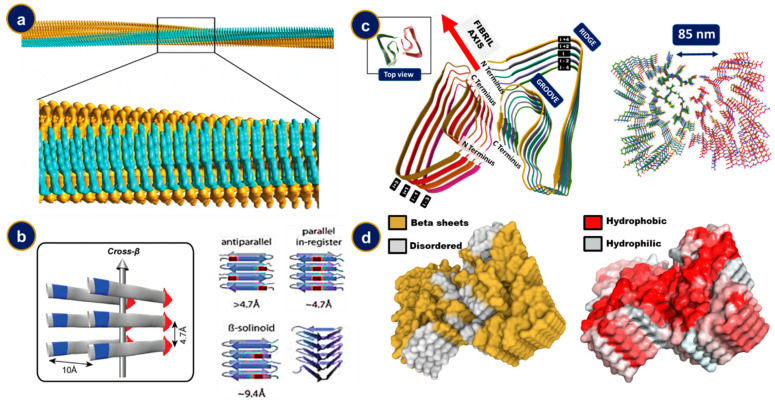
The structural features of amyloid fibrils: (**a**) 3D map of ATTR amyloid fibril constructed from cryo-EM image. Cyan depicts the N-terminus residue density and ochre depicts the C-terminus residue density (figure reproduced from [52]); (**b**) typical cross-beta-sheet motifs found in amyloids (the figure reproduced from [53]); (**c**) top and side views of the cartoon depiction of an amyloid fibril fragment showing the non-planar sub-units in a staggered conformation. The top view depicts a dry interface devoid of water formed due to interdigitation of the side chains of residues on opposite chains; (**d**) secondary structure and hydrophobicity mappings on the amyloid beta fibrils. The hydrophobic interactions in the core (red) bear testimony to its exceptional thermodynamic stability.

Amyloid depositions are found in various morphologies of which the meshed, fibril bundles and the star-shaped supercluster are the most common. While the fibrillar bundles are compact unidirectional deposits, the mesh morphology lacks a definite orientation and the star-shaped cluster consists of supercoils radiating in multiple directions [40,41]. Individual strands exist in a fibrillar, unbranched, thread-like state, generally 7–13 nm in diameter and a few micrometers in length [42]. The fibrils, in turn, comprise of 2–8 protofilaments which are 2–7 nm in diameter. These protofilaments may either associate in a twisted way with one another or laterally as flat ribbons of thickness 2–7 nm and about 30 nm wide [43]. The protofilaments possess an extended beta-sheet secondary structure with the individual beta-sheets being perpendicular to the longitudinal fiber axis, a structure referred to as the cross beta [44,45,46] (Figure 2). The etymology of the rather colloquial term now lies in the characteristic diffraction data of these fibrils where two distinct diffraction line sets at 0.47 nm and 1.0 nm correspond to the longitudinal inter-strand transverse stacking distances [47]. However, it is worthwhile to note that only a small percentage of the polypeptide stretch forms these beta associations while the remaining portions assemble as loops, tails, and/or intrinsically disordered structures [48,49,50]. The side chains in the residues forming the beta sheet assemblies interdigitate to form a hydrophobic water devoid region referred to as the dry region and constitute the steric zippers (pairs of self-complementary β-sheets) [51]. This water extrusion phenomenon upon amyloid formation drives the entropic gain for facilitating the aggregation.

The constituting β sheets may further fold upon themselves, leading to a great deal of polymorphism in amyloid fibrils. Both electrostatic and Van der Waals interactions, along with the contiguous array of hydrogen bonded residues, have an essential effect in attributing structural stability to these fibrils. Recent computational efforts further shed light on the importance of these non-bonding interactions in imparting mechanical properties, such as elasticity and thermodynamic stability, to these self-assemblies [54,55]. Often, two β-sheets come face to face, separated only by a loop–hinge region forming a structure termed as β sandwich [56]. An iterative pattern of the same kind leads to the formation of a supersecondary structural motif called superpleated β-sheet, where the individual β-sheets align parallel to each other. β-sheets can be in-register, when residues on one strand align with the same residue on the other strand, or out of register, when residues on one strand do not align with the same residues on the other strand [57,58]. Another intriguing motif prevalent in the amyloid family is that of β solenoids. In line with the etymology, it consists of an array of a polypeptide chain looping back on itself to form another layer. Clearly, this structure cannot be in-register. However, if the adjacent beta strands show a great deal of sequence similarity, it is termed pseudo-in-register. The β-solenoid, in turn, also encompasses a wide variety of structures classified on how the β strands coil around the central imaginary axis. The most notable ones prevalent in amyloid fibrils include β-helix, where the individual strands associate in a helical fashion with either two or three faces, and β-roll, consisting of two β-strands looping in a β-sandwiched fashion to form another identical layer [59]. Various computational as well as experimental efforts toward mapping the electrostatic surface potentials have bettered the understanding of formation, and shed light on the heterogeneity of these deposits [60].

## 5. Characterization of Amyloid Fibril Formation

Protein misfolding, aggregation and fibril formation have emerged as important areas in neurodegenerative diseases and recombinant biopharmaceutical products. Accordingly, different classical and updated biophysical techniques have been utilized to investigate the aggregation of proteins in vitro and in vivo. For example, the secondary structure of amyloidogenic proteins can be investigated using circular dichroism spectroscopy (CD), fibrils formation can be studied using molecular probes, and the morphology of the aggregation can be investigated via transmission electron microscopy (TEM) [61,62,63].

**Figure 3:**
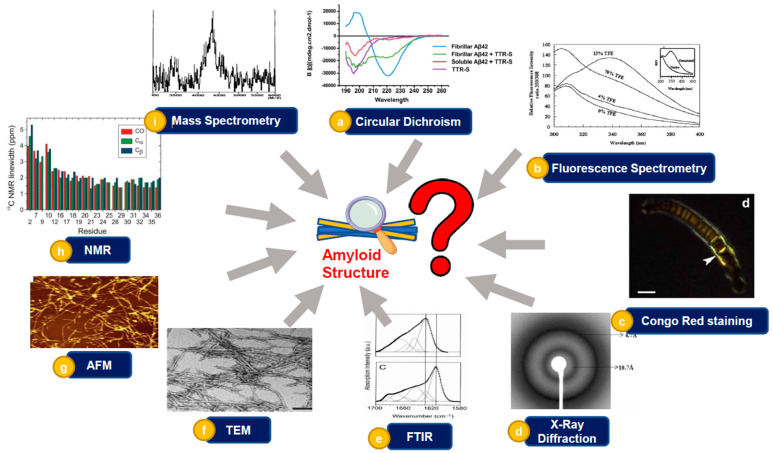
Common biophysical techniques used in the characterization of amyloid fibrils: (**a**): CD spectra of Aβ 42 fibrils in presence and absence of TTR (figure adopted from [67]; (**b**): the intrinsic fluorescence of nFGF-1 in various concentrations of TFE (the figure was adopted from reference [71]; (**c**): staining with red Congo of plant (Prasiola linearis adhesive) (the figure adopted from [76]); (**d**): the X-ray fiber diffraction of the n-FGF1 fibrils induced by TFE (the figure was adopted from reference [77]); (**e**): FTIR for native (1) and (2) fibrils TTR (the figure was adopted from [97]; (**f**): TEM of fibrils extracted from heart tissue samples (the figure was adopted from reference [89]); (**g**): AFM of Aβ 1–40: aggregation of Aβ-1–40 (the figure was adopted from [92]; (**h**): the linewidths of 13C NMR for CO, Cα, and Cβ location in Aβ1–40 fibrils (the figure was adopted from [94]); (**i**): analysis of cerebral cortex Aβ by mass spectrometry. The broad peak reveals the non-homogenous shape of the protein (figure was adopted from reference [95]).

Secondary structures play a vital role in the aggregation process, and hence, knowledge about the secondary structure and perturbations to it is of paramount importance. For instance, CD analysis shows that unordered and α-helical structures are the main content of the monomers and oligomers of Aβ deposits [64,65,66]. CD spectroscopy has been employed in investigating the effect of transthyretin (TTR) on the structure of β-amyloid that causes cellular toxicity [67]. The structural characterization of Aβ_42_ amorphous aggregates using CD spectroscopy revealed that the addition of TTR-S (sequence YTIAALLSPYSYSRRRRR) to preformed fibrils caused a significant change in the secondary structure of Aβ_42_ from β-sheet to helical secondary structures that leads to protection against cytotoxicity (Figure 3a). Analysis of the tertiary structure of proteins by monitoring the changes in intrinsic fluorescence or the intensity of fluorescence of a specific dye, such as Thioflavin T (ThT), which can bind to β-sheet aggregates and increase fluorescence intensity significantly as it binds to more aggregates, has been used widely used in detection of the fibrils formation [68,69]. In a study that examined the structural changes of newt acidic fibroblast growth factor (nFGF-1) in the presence of the 2,2,2-trifluoroethanol (TFE), the fluorescence spectra of newt acidic fibroblast factor (nFGF-1) were investigated, and no change in the spectrum of the protein was observed at lower concentrations of TFE through mentoring the intensity of fluorescence at wavelength 350 to 308 nm. The fluorescence spectra of nFGF-1 exhibits an emission maximum at around 308 nm when the protein is folded because the fluorescence of the tryptophan residues are quenched by imidazole and pyrrole groups in the environment of the indole ring of the tryptophan residues [70]. However, at higher concentration of TFE (8 to 15% (*v/v*)), the ratio of the 350 to 308 nm fluorescence significantly increases, indicating significant change in the tertiary structure of the protein. The most significant effect of TFE on the structure of nFGF-1 was found at 15% (*v/v*) TFE (Figure 3b) [71]. Extrinsic fluorescence dependent on staining dye is also employed in fibrils formation analysis [72]. Thioflavin T (ThT) is a small molecule dye, whose derivatives are widely used to detect the aggregation of amyloidogenic proteins. ThT exhibits fluorescence emission maximum at around 485 nm wavelength due to its binding to the amyloid. It has been indicated that ThT binds to prefibrillar aggregates that contain the β-sheet groove binding site. According to the study, the binding site of prefibrillar aggregates of the β-sheet structure has lower (ThT) fluorescence intensity than fibrils. However, the (ThT) fluorescence of the prefibrillar species could not be detected in the study [68,73,74]. Besides extrinsic fluorescence, another staining method has been used in the amyloid analysis employing Congo red (CR). It has indicated that CR assisted in detecting amyloid fibrils in the form of deposits in the brain tissue and in vitro. During the investigation of the possible formation of amyloid structures in algal adhesive, the green–gold birefringence was observed after staining with Congo red [72,75,76] (Figure 3c). CR binds to the β-sheet structure found in amyloid fibrils and detects a significant change in the maximum absorbance from 490 to 540 nm and green birefringence with blended polarized light. 1-anilinonaphthalene 8-sulfonate (ANS), and 4,4′-bis-1-anilinonaphthalene 8-sulfonate (Bis-ANS), are fluorescent dyes incorporated in protein characterization since the 1950s, and they are applicable in the detection of protein aggregation [62,74]. The knowledge about the exposed hydrophobic patches in protein can be achieved via ANS binding assay when the fluorescence intensity is elevated, and the fluorescence maximum has blue shift due to the exposure of the hydrophobic parts on the surface of proteins [77].

Another stain that is frequently used in the investigation of amyloid fibrils is Congo red (CR). The dye binds to the β-sheet structure found in amyloid fibrils and detects a significant change in the highest absorbance from 490 to 540 nm and green birefringence with blended polarized light [78,79]. It has been demonstrated that CR detects amyloid fibrils in brain tissue [80]. Furthermore, in algal adhesive, the green–gold birefringence was observed after staining with CR indicating amyloid formation (Figure 3c).

X-ray diffraction has been used as a powerful and sensitive technique providing information about the structure and assembly of amyloid fibrils. For instance, it has been observed that the aggregation of newt protein acidic fibroblast factor (nFGF-1) induced by 2,2,2, trifluoroethanol (TFE) exhibits reflection mainly at 4.7 Å on the meridian and identical equatorial reflection at 10.3 Å, which indicates that the fibrils induced by TFE possess a cross β structure (Figure 3d) [81].

Fourier transform infrared spectroscopy (FTIR) is a nondestructive technique which has been widely used in the analysis of protein and peptide structure. Amide I and amide II are the two major bands investigated via FTIR. Amide I is primarily associated with the stretching vibration of C=O, while amide II is related to the bending of N-H and stretching of C-N vibrations of the peptide structure [82]. The frequency of the amide I band is mainly related to secondary structures of the protein and is based on various hydrogen-bonding interactions in the α-helix, β-sheet, β-turn, and unordered structures. The amide I vibrational frequencies of α-helices and β-sheets are about 1655 and 1630 cm^−1^, respectively, while the vibrational frequency of the same for the aggregated protein is about 1620–1625 cm^−1^ [83,84,85]. Native transthyretin (TTR) shows an absorption spectrum that has a wide amide I band with a maximum at 1630 cm^−1^, while (TTR) fibrils display a spectrum with an amide I band with a maximum at 1615 cm^−1^ and an additional low-intensity peak at 1684 cm^−1^ [86] (Figure 3e). Furthermore, FTIR can be employed to investigate protein misfolding and aggregation in cells and tissues and picture the in situ secondary structure of the amyloid plaques in brain tissue of AD patients using synchrotron Fourier transform infrared micro spectroscopy (FTIRM) [85,87].

Morphology analysis has emerged as an essential technique in investigating amyloid formation. Among the highest resolution microscopic techniques, transmission electron microscopy (TEM) and atomic force microscopy (AFM) have most widely been used for observing the morphology and structure of macromolecular assemblies involved in the pathogenesis of protein-misfolding diseases [88]. In the TEM technique, a cathode ray emitter is utilized to provide and expedite a high-voltage electron beam focused via electrostatic and electromagnetic lenses. While the electrons move through a thin and electron-transparent sample, an image is produced from the electrons transmitted over the sample. Then, the image is magnified and focused via a detached lens, which can then be displayed on an imaging screen [88]. Several pieces of information related to the structure of the sample can be inferred from the image. Then, the image is amplified via electromagnetic lenses and measured by a fluorescent screen, photographic plate, or a charge-coupled apparatus. Usually, macromolecular minerals are set onto a fine, metallic grid support covered by a thin, electron-transparent carbon film. Negative staining, rotary shadowing, and cryo-EM are helpful to negatively stain biological macromolecules to increase the comparison and decrease the image noise. TEM pictures provide visualization of the biological macromolecules in vitro and in vivo. For example, fibrils purified from the heart of a 51-year-old woman with light chain (AL) amyloidosis were visible through TEM under negative staining conditions [89] (Figure 3f). Using atomic force microscopy (AFM), a dynamic technique indeed, different kinds of surfaces can be visualized, encompassing, but not limited to, polymers, ceramics, composites, glass, and biological samples. The technique has been indicated as an important one in amyloid analysis, and it has been widely used to describe the morphology, assembly progress, and mechanical properties of amyloids [90,91]. There exist mainly two major techniques to use AFM. In the first technique, the needle tip is constantly attached to the sample and is referred to as the contact mode. In contrast, in the tapping mode, a rigid girder oscillates and can be close to the specimen, while another component of the oscillation expands into the abhorrent reign. Therefore, it occasionally connects the edge and the surface. In a study of self-aggregation of β-amyloid fibrils utilizing tapping mode atomic force microscopy, after one week of incubation, it was found that the fibrils of Aβ_1–40_ had high surface density and extended length (5.1 μm) (Figure 3g) [92].

Solid state nuclear magnetic resonance spectroscopy (ss NMR) has become one of the best choices for amyloid structure determination among different resonance techniques, and it is suitable for solid or solid-like samples for various reasons. Amyloid fibrils produce high quality results with this technique, because they have a recognized molecular structure, can be prepared with selective isotopic labeling in multi-milligram amounts, and in high concentrations, which yields a good signal to noise ratio as a result [93]. In a study describing a molecular sample for Aβ_1–40_ fibrils via solid state NMR and other experimental results, the N-terminus region of Aβ_1–40_ showed deranged fibrils for the entire structural arrangement, starting beyond Y10 (Figure 3h) [94]. 

Mass spectrometry, a well-known analytical technique, can detect the mass/charge value of the protein fragments. In addition, the technique has exhibited a vital role in detecting and characterizing proteins aggregation and fibrils formation. For instance, Aβ purified by two-dimensional reverse-phase high pressure liquid chromatography (HPLC) was investigated via amino acid sequencing and mass spectrometry after treatment with a lysylendopeptidase. The study revealed a vast peak that distributed asymmetrically around a mass of 4356.7, indicating that Aβ protein was not homogeneous [95] (Figure 3i). Analysis of the self-assembly of insulin via mass spectrometry is another example where the self-assembly and aggregation of insulin molecules were analyzed using nanoflow electrospray mass spectrometry [96].

## 6. Amyloid Precursor Protein and Its Involvement in Amyloid Formation

Amyloid precursor protein (APP) is a type 1 single-pass transmembrane glycoprotein, and one of its products of metabolism is the Aβ peptide [98,99]. The sequential proteolytic cleavages in APP lead to the generation of β-amyloid peptides (Aβ) [99]. APP is comprised of three domains: APP extracellular domain, APP transmembrane domain, and APP intracellular domain, and has three most common isoforms: APP_695_, APP_751_ and APP_770_ [100]. The entire structure of the APP is yet to be ascertained. However, the crystal structures of the E1, E2 subdomains, and the ACID domain are known [101,102]. The N-terminus, extracellular domain 1 of APP (E1 region) includes a growth factor-like domain (GFLD) and a copper-binding domain (CuBD) [103,104]. The extracellular domain 2 (E2 region), which is a linked linker, contains the RERMS sequence and the central APP domain [103]. The intracellular region of APP, called the APP intracellular C-terminus domain (AICD), is known for two proposed functions, as a transcriptional regulator and in regulation of its trafficking and endocytosis with the YENPTY motif [105,106].

## 7. APP Processing Pathways

Pathogenic mutations in APP lead to a surge in Aβ peptide production and/or change in the ratio of certain Aβ peptides, and thus, have been linked to the early onset of AD [101,105,106]. APP processing occurs via the action of secretases. This leads to two processing pathways: amyloidogenic and non-amyloidogenic [107,108]. In the amyloidogenic pathway, APP is cleaved by β-secretase (BACE1 and BACE2) and releases soluble secreted APP (APPsβ), and membrane-tethered-β-carboxyl-terminal fragments (CTFβ) [109,110,111]. The γ-secretase then cleaves CTFβ with an endoproteolytic cut at the ε-site liberating ACID, and an exoproteolytic cut at the N-terminus generating Aβ [101,112,113]. On the other hand, in the non-amyloidogenic pathway, APP is cleaved by α-secretase to release APPsα and membrane-tethered α-carboxyl-terminal fragments (CTFα); γ-secretase then cleaves the latter to generate ACID and p3 [99,107,114]. Curiously, until 1992 it was believed that the cleavage of the CTF-β by γ-secretase required some membrane perturbation since the proteolytic cut had to occur at the transmembrane site. Therefore, it was postulated that following the β-secretase cleavage, a cleavage could occur at COOH terminus of the Aβ region, which would be facilitated by γ-secretase [115]. 

**Figure 4:**
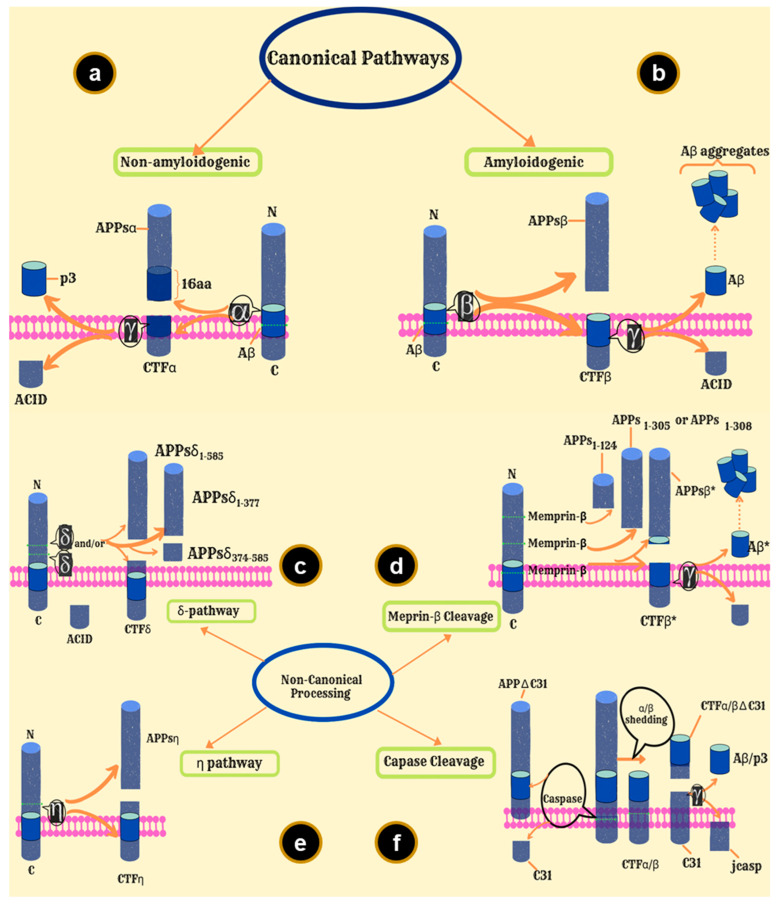
The canonical amyloid precursor protein (APP) processing pathways: (**a**) processing by α-secretase along the non-amyloidogenic pathway occurs in the amyloid-β (Aβ) region, liberates APPsα (α-secretase-generated APP ectodomain fragment) and generates p3; (**b**) processing along the amyloidogenic pathway generates Aβ (through β-secretase and γ-secretase cleavage) and liberates APPsβ. The schematic shows the non-canonical APP processing; (**c**) cleavage by δ-secretase produces three soluble APPsδ fragments and C-terminal fragment-δ (CTFδ), which are further processed by β-secretase and γ-secretase; (**d**) cleavage by meprin-β at three sites gives rise to three soluble fragments (top right panel). APPsβ* contains one additional residue compared with APPsβ; (**e**) cleavage by η-secretase gives rise to soluble APPsη and CTFη, which is further processed by α-secretase or β-secretase to generate Aη-α or Aη-β; (**f**) caspases cleave within the intracellular domain to yield C31 and after subsequent γ-secretase cleavage.

The amyloidogenic and non-amyloidogenic pathways are canonical pathways. There exist several non-canonical APP processing pathways [116]. In the η-secretase pathway, APP is cleaved within the extracellular domain releasing sAPP-η and C-terminal fragments (CTF-η) [117]. CTF-η is then processed either by α-secretase releasing the Aη-α peptide (leaving a CTF-α fragment) or by β-secretase releasing the Aη-β peptide (leaving a CTF-β), to the extracellular medium [118]. Meprin-β can cleave all APP isoforms at multiple sites on the N-terminal ectodomain, one of which releases APPsβ*, and the C-terminal ectodomain liberates CTFβ* [119,120]. CTFβ* is one residue shorter than CTFβ [101]. CTFβ is then processed by γ-secretase liberating Aβ* and AICD [121]. Aβ* is amino-terminally truncated Aβ_2−x_ [101]. The APP C-terminus can also be cleaved by Asp664 to release [122]. In the caspase APP processing pathway, a small peptide, Jcasp, is generated by the cleavage of CTFs by caspase and γ-secretase [123,124]. In the δ-secretase pathway, three soluble APPsδ fragments and C-terminal fragment-δ (CTFδ) are produced upon cleavage by δ-secretase, which are then processed by β-secretase and γ-secretase, liberating Aβ and AICD [101,125] (Figure 4).

## 8. The Aβ Peptide

The exoproteolytic cut at the N-terminus (Aspartyl) of the membrane tethered 99 residues long CTF β by γ secretase is not precise [121,126]. Consequently, the resulting Aβ peptide shows heterogeneity in residue length and C-terminus polymorphism. The C-terminus of the peptide plays a pivotal role in the alpha helix to beta sheet conformational change during aggregation processes. Amidst the pool of various fragment lengths of these Aβ peptides, ranging from 37 to 51 residues, the 40-residue one is most abundant, followed by the 42 residues [3,19,20], the peptides denoted as Aβ_40_ and Aβ_42_, respectively [42,127,128]. These are formed from the larger Aβ fragments by endocytic processing [129,130]. The Aβ_42_ variant has been found to be more hydrophobic and has a higher amyloidogenic propensity than that of the Aβ_40_ counterpart [131,132,133]. The longer Aβ_42_ isoform of the peptide is formed in the Endoplasmic Reticulum (ER), while the shorter Aβ_40_ is formed in the trans-Golgi network [134,135]. 

Neprilysin (NEP), Endothelin Converting Enzymes (ECE 1&2), Matrix Metalloproteases (MMP 2) and Insulin Degrading Enzyme (IDE) are the principal metalloproteases responsible for the catabolism of Aβ peptides [136,137,138,139]. Aβ peptides also undergo lysosome mediated degradation, involving enzymes such as cathepsin B [140]. Plasmin, a serine protease, has also been found to have a degrading effect on both Aβ monomers and fibrils [141]. AD is often associated with mutations and/or underproduction of these enzymes, leading to decreased metabolism of Aβ peptides. Soluble Aβ fragments can cross the blood brain barrier to fall into the mainstream blood circulation [142]. This exchange is mediated by Low Density Lipoprotein (LDLP) receptor related proteins (LRPs) on the brain side and the receptor for advanced glycation end products (RAGE) on the luminal side [143,144]. Modifications in these vascular mechanisms might additionally contribute to increases in concentrations of Aβ fragments in the brain in AD related pathologies.

**Figure 5:**
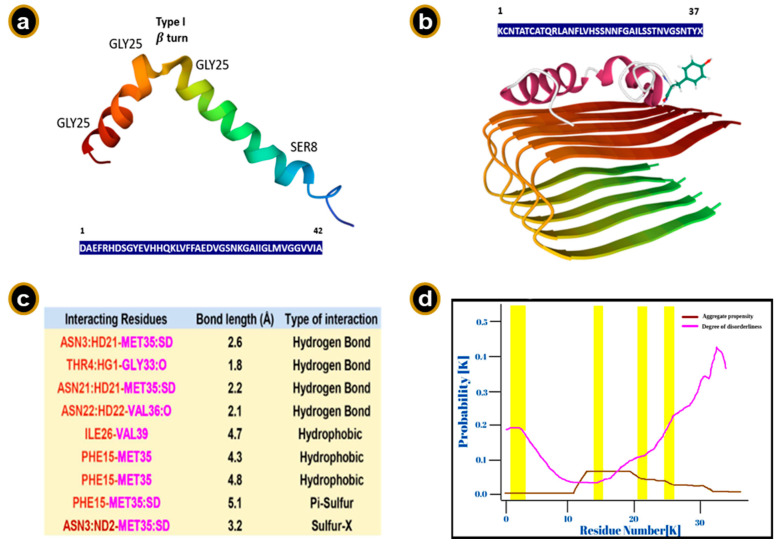
(**a**) Structure and sequence of human Aβ peptide; (**b**) amylin binds with Aβ peptide through its various interacting residues, resulting in a strong association (binding affinity energy = −8.83 kcal/mol; (**c**) possible interacting residues of human amylin-red (PDB ID: 2L86) and β sheet structure of Aβ1–42-magenta (PDB ID:2BEG); (**d**) computational analysis of disorder (red curve) and aggregation promoting(magenta) regions on human amylin-red (PDB ID: 2L86) upon binding with interacting residues (yellow) of Aβ1–42 (PDB ID:2BEG). (Figure b–d were reproduced from [151]).

The structure of the Aβ peptide is dynamic in solution and depends on various factors, such as polarity, etc. The different conformations have been shown to exhibit varied propensities towards the amyloid formation. Moreover, different conformations of the monomer may favor different aggregation pathways, leading to varied kinetics in solution [145]. Aβ peptide is mainly α helical in organic solvents, whereas it assumes predominantly a β sheet structure in aqueous media, albeit subject to pH, concentration and incubation times [146,147]. In aqueous sodium dodecyl sulfate (SDS) media, the Aβ_40_ peptide between residues 1–14 is unstructured, the residues being polar and likely been solvated by water. The Aβ_40_ peptide has been shown to possess an α helical conformation at the C-terminus between residues 15–36 with a kink or hinge at residues 25–27 and a bend at residue 12 in membrane mimicking solutions. His-13 and Lys-16 have their sidechains lying close on the same side of the helix and are in close proximity. Moreover, 3D NMR studies report two helical regions, Aβ (8–25) and Aβ (28–38), being connected by a type I β turn (Figure 5a). The C-terminus of the Aβ_42_ isoform is more structured and has reduced flexibility due to a β hairpin structure formed by residues 31–34 and 38–41, probably attributing to its greater amyloidogenic propensity over the Aβ_40_ isoform. Recent studies show that the Aβ peptides can populate a multitude of discrete conformational clusters [148]. Furthermore, recent computational and experimental efforts reveal that the association of N-terminal hexapeptide A2T and A2V with human Aβ_42_ shows significantly lower amyloidogenic propensity by altering the conformational landscape of human Aβ_42_ [149,150].

Protein docking studies between Aβ peptides and human amylin reveal that the major interactions are in the intrinsically disordered stretches of the proteins, suggesting that amyloid aggregation could be driven by the disordered regions of these peptides (Figure 5b–d). Furthermore, these feasible interactions also hint at explaining the co-occurrence of AD, PD, and type-II diabetes [152]. Roughly, a quarter of the Aβ peptide surface is hydrophobic [153]. α helix to β sheet transition of the peptide is thought to involve deprotonation of side chains of the acidic residues Asp7, Glu11, Glu 22 and Asp23 at a pH greater than 4 and the subsequent protonation of the basic residues His6, His 13 and His 14. This event causes a disruption of the α helical structure and above a critical concentration leads to the formation of an oligomeric β sheet structure and fibrillization. Salt bridge electrostatic interactions between His-Asp/Glu residues and the hydrophobic Van der Waals interactions in Leu17-Ala21 residues play a major role in stabilizing the antiparallel arrangement of β sheet assembly defined by residues 13–23. We shall delve into an in-depth discussion about the self-assembly process of amyloid fibrils in the subsequent section(s) [25]. 

## 9. Sequence Requirements

Interestingly, even shorter fragments of the Aβ peptides are also capable of forming amyloid fibrils. Norstedt et al. proposed that the shortest fragment capable of amyloid formation is the decapeptide Aβ 14–23 [154]. Any mutations and/or substitutions in this region of the Aβ peptide have been shown to be generally associated with loss of fibril forming ability, indicating that this sequence forms the core of the Aβ fibrils. Almost a year later Tycko et al. showed that the hepta residue Aβ16–22: N-acetyl-Lys-Leu-Val-Phe-Phe-Ala-Glu-NH2, formed full length and highly ordered amyloid fibrils in vitro [155]. Substitutions for positions that stabilize the α-helical conformation (for instance, substituting Val18 by Ala) have been shown to reduce the amyloidogenic propensity of the peptides [156]. It is indeed intriguing that even single point substitutions can act as a veto against fibrillation. For instance, any substitution for Lys16 or Phe19/20 by Ala has been shown to block fibril formation in vitro [157,158,159,160]. Certain mutations indicate that induction of aromatic amino acids increase the amyloidogenic propensities, whereas some studies claim that mutations which replace aromatic side chains promote aggregation of the Aβ peptide, negating the role of π stacking in amyloid self-assembly [22,161]. Concurrently, there exist key regions where substitutions can enhance amyloidogenic propensities [162,163]. These substitutions enhance the hydrophobic interactions in the hydrophobic core by decreasing the electrostatic potential in the 17–21 region [164]. The residues 1–9 of Aβ N-terminus are exposed on the fibrillar surface and play a role in additionally stabilizing the fibrils via interfibrillar interactions. However, Aβ peptides with 1–9 excised region have been shown to form fibrils, indicating that residues 1–9 are not necessary for fibril formation [165,166].

## 10. Conservation of the Sequence

**Figure 6:**
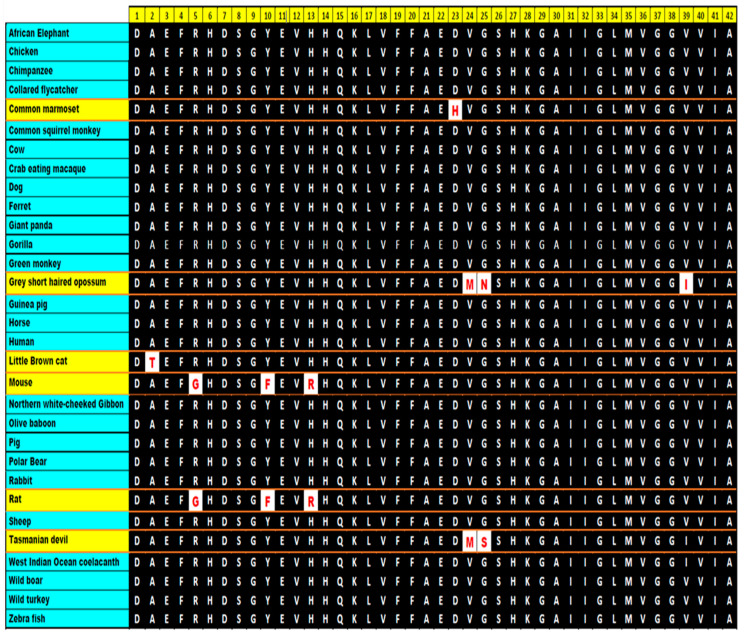
Primary sequence of the Aβ peptide in different organisms arranged in lexicographical order. The sequence is highly conserved except for rare point amino acid mutations in some organisms.

The Aβ peptide is not exclusive to humans. It occurs in a large variety of organisms, however, pathologies related to AD-type diseases are almost exclusively observed in humans alone [167]. The Aβ peptide sequence is highly conserved with only some point mutations in some species (Figure 6). Apart from chimpanzees, almost all non-human primates lack the typical neurofibrillary tangle pathology—a hallmark of AD, even though the NHPs have almost similar APP sequence and biochemistry in addition to Aβ sequence similarity [168]. Interestingly, there has been evidence that the soluble Aβ content in chimpanzees might be higher than those in clinical AD cases [169]. Canines, although genetically farther from humans than non-human primates (NHPs), exhibit substantial amounts of Aβ_42_ triggered amyloid depositions related to cognitive dysregulations. Moreover, the amyloid buildup starts much faster in canines compared to NHPs. However, the pathologies exhibit little or no neuronal loss. They also lack neuritic plaques or neurofibrillary tangle pathology, another point of contrast between AD in humans and age-related amyloid pathology in canines [170,171,172] (Figure 6). 

Now, does it mean that amyloid formation is driven by a specific primary sequence or is it more generally an inherent property of polypeptides under a given biochemical environment? In this context, an interesting observation was reported by Guijarro et al., wherein they found PI3-SH3 small globular domain with a simple native fold formed amyloid-like aggregates at acidic pH, whereas at neutral pH no amyloid formation was observed [173,174,175,176].

A year later, Chiti et al. showed that acylphosphatase with very little propensity to adopt β sheet structure even in the partially folded state, could be directed to form amyloid-like aggregates in vitro. It clearly hinted that, probably, the driving criterion was not entirely the high propensity primary sequence but rather the correct denaturing conditions favoring the hydrophobic clumping forming aggregates [177]. Subsequently, it was also shown that homomeric polypeptides, i.e., polypeptides containing residues of same amino acids, especially those of aromatic amino acids, such as polylysine and polythreonine, could form amyloid-like fibrils [178]. More recently, it has also been shown that single amino acids, such as phenylalanine, can form amyloid assemblies, thus strengthening the hypothesis [179].

## 11. Protein Aggregation and Its Mechanisms

**Figure 7:**
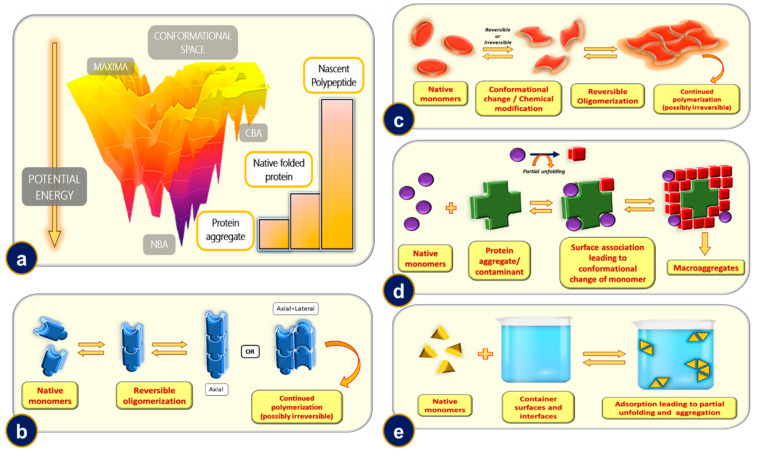
Aggregation mechanisms: (**a**) the potential energy surface for a protein folding process. The competing basins of attractions are populated by the folding intermediates which have higher energy than the native folded protein. The native basin of attraction is a low-lying critical point, populated by the functionally folded native protein. The aggregated being thermodynamically stabler lie even more downhill, populating the lowest points of the PES; (**b**) various mechanisms for modeling protein aggregation: reversible oligomerization of the native monomers leading to irreversible macroaggregates; (**c**) conformational change of native monomer followed by b1 mechanism; (**d**) microaggregate/contaminant induced surface aggregation leading to irreversible polymerization and aggregation; (**e**) aggregation induced at phase interface or on rough surfaces.

Proteins are the true “chameleons” when it comes to structural diversity. The same protein exists in markedly different structural forms depending on the chemical, physical and biological environments. It is a well-imbibed fact that the proteins keep folding up on themselves to gain stability, giving rise to various levels of structures: primary, secondary, tertiary, and quaternary. Protein folding, which is ubiquitous in nature, is a rather complex problem when it comes to predicting the final state starting from the primary structure, colloquially termed as the protein folding problem [180]. A rather appealing and comprehensive way of understanding the relative stabilities of the sibling structures of the same protein is by analyzing the potential energy surface diagram of the same, more well known as the folding funnel diagram [181]. The folding funnel is a multidimensional potential energy landscape which correlates various structures and/or levels of protein organization with their relative stabilities. The nascent or the unfolded protein chain (primary structure) occupies an intermediate energy level. The local minimas, also known as competing basins of attractions (CBAs), are generally populated by the folding intermediates and the misfolded protein structures. The global minima(s) or the native basins of attraction (NBAs) are populated by the most stable protein structures, which include the native folded state and aggregates of proteins. The topology of the energy landscape reveals yet another interesting aspect of the protein folding process. The folding funnel can either be a smooth one or a rugged one. The rugged funnel is generally an indication that molecular chaperones assist in the folding of the protein under consideration [182] (Figure 7a).

A smooth funnel scenario arises for small globular proteins where the native folded state is determined by the primary sequence alone. The most well-known example in this context is probably the case of ribonuclease folding, enunciated by the 1972 Nobel Laureate in Chemistry—Christian B. Anfinsen—in the principle now known as the Anfinsen’s dogma. Anfinsen, in his studies of ribonuclease folding, had postulated that a primary protein sequence could be used to predict the final folded structure of a protein uniquely, given that the native state minima in the potential energy surface (PES) is unchallenged by any other comparative structure of the protein, and the path leading from unfolded to folded state is kinetically and thermodynamically accessible without having to go through complex conformational requirements [183,184]. It is interesting to note that amyloids—our current topic of interest—are an exception to this dogma as they represent an additional minimum other than that of the native state of the protein in the folding funnel. Protein structure is highly dynamic, and temperature-induced conformational fluctuations lead to a sparse population of partial/misfolded states in the Boltzmannian ensemble [185]. Some of these structures have aggregation-prone residues exposed on the surface, and these structures contribute largely to initiate the aggregation cascade. It is however not necessary that these aggregation prone structures should always originate from the native state and can trace back their origin to pre-native form(s) of the protein. Protein–protein interactions are of utmost importance to probe aggregation mechanisms. While electrostatic interactions between protein monomers/oligomers have been shown to promote aggregation, the role of hydrophobic interactions in the same remains debatable. While one school of thought advocates that since introduction of hydrophobic amino acids at aggregation hotspots increases the aggregation propensity, hydrophobic interactions must be the major driving force behind protein aggregation; another school of thought argues that the hydrophobic patches occupy too small a region on the exposed protein surface to be able to mediate hydrophobic aggregation [186,187,188].

Protein aggregation is primarily entropy driven. Superficially it might seem that the process is entropically disfavored as aggregates should have lower entropy than free monomers/oligomers; however, this decrease is compensated more than enough by the entropy increase due to water molecules being extruded from aggregating regions of the interacting proteins. The overall entropy change for the process thus becomes positive [189]. While several mechanisms have been proposed for an overwhelmingly large statistical sample of proteins, almost all of them have a common skeletal framework. The initiation of protein aggregation takes place by formation and/or introduction of a nucleating center, which would drive forward the aggregation. We have tried to elucidate some simple aggregation models which can describe a fairly large number of protein aggregation pathways. 

### 11.1. Mechanism-1: Reversible Oligomerization of Native Monomers

For proteins that have aggregation-prone residues exposed on the surface, the monomers may possess an intrinsic nature to oligomerize albeit reversibly until the formation of a critical nucleus size. It is interesting to note that in most cases the kinetic data suggest that k_off_ (rate corresponding to monomer detachment from forming oligomer) is greater than k_on_ (rate corresponding to the addition of a monomer to the forming oligomer) until the formation of a critical nucleus, after which the rate correlation reverses, thus favoring aggregation [189]. The formation of the critical nucleus might be explained based on stochastic fluctuations in the system. The forming nucleus may even catalyze the formation of another potential nucleus on its elongating surface, a process termed secondary nucleation. The larger oligomers over time thus have a much lower tendency to dissociate into native monomers, and the oligomerization eventually becomes irreversible. Depolymerization/oligomerization can also be inhibited in cases where the protein monomers associate chemically (for, e.g., via disulfide bridges). Insulin and Interleukin-1 receptor antagonist (rhIL-1RA) proteins are probably the best examples that fit this mechanistic model [190,191] (Figure 7b).

### 11.2. Mechanism-2: Aggregation of Conformationally/Chemically Modified Monomers

As discussed earlier, proteins populate an ensemble of structures given approximately by the Boltzmannian distribution. At physiological temperatures, although there might be negligible populations of mis/partially folded states with respect to the native form, with increasing temperature their population increases. These conformationally distinct monomers can then follow a mechanism as outlined in Mechanism-1 (reversible oligomerization). This initial change in the native monomer might even be brought about by chemicals (not necessarily denaturants). For example, proteolysis of a short stretch of polypeptide or glycosylation, changes brought about by pH might alter the aggregation propensity of the protein molecules [192,193,194]. The conformational and/or chemical changes in the native monomer might even be brought about by physical cues, such as pressure, etc. [195]. After the formation of a critical nucleus, it is possible that even the native monomers are recruited into the aggregating polymer. IFN-γ and G-CSF are reportedly thought to undergo aggregation via this mechanism [196,197,198] (Figure 7c). 

### 11.3. Mechanism-3: Microaggregate and/or Contaminant Induced Aggregation

Nucleation as discussed is a critical step in aggregation. Expectedly, introduction of a nucleus of suitable size expedites the aggregation process. To draw an analogy, it is equivalent to the introduction of a seed in crystal solution to initiate and expedite crystallization. When the nucleus introduced, as an oligomer of the aggregating protein, it is termed as a homonucleating process, whereas when aggregation is induced by some external particle (aggregate of a different protein or rough micro beads), it is termed as heteronucleation. The introduction of a nucleus of a proper critical size (or larger) may immediately lead to an aggregation cascade while any nuclei of a size smaller than the critical size would be slower at inducing aggregation until it grows to reach the critical size [199,200,201,202] (Figure 7d).

### 11.4. Mechanism-4: Phase Interface or Rough Surface Induced Aggregation

At phase interface or on rough surfaces, the native monomers may undergo adsorption induced conformational change and/or partial unfolding. This event may trigger the formation of a critical nucleus at the surface/phase interface, mediating towards aggregation via the above-mentioned mechanism(s) [203,204,205]. In such cases, surfactants may reduce the aggregation by minimizing exposed surface area of the native monomers or that of the nucleus, thereby arresting oligomerization to a large extent. It should be noted that this bifurcation of mechanisms is purely artificial, and a given protein may aggregate through one or more of the mechanisms we highlighted [206,207] (Figure 7e). 

## 12. Kinetics and Mechanisms of Amyloid Formation

Amyloids differ from amorphous protein deposits in the fact that they possess a highly ordered fibrillar morphology. It is worthwhile to discuss the kinetics alongside the various mechanisms that contribute to amyloid formation. For the purpose of enhanced readability of the present article, we have tried to keep the mathematical treatment to the bare minimum without compromising the vigor of the kinetic revelations. One of the first attempts to model kinetics of filamentous protein aggregation can be traced back to the 1960s, wherein Ossawa et al. modelled the polymerization of G-Actin to form filamentous F-Actin [208]. They showed that the dynamic length of an actin filament depended on the concentration of the free monomers as well as concentration of the existing fibrils along with distinct rate constants of polymerization and depolymerization [209].

This set the ground for the development of several primary NDP (nucleation dependent polymerization) models. There exist various flavors to these NDP models, however the basic essence revolves around formation of a nucleus of a critical size in a thermodynamically unfavored (but not stochastically impossible) process after which addition of monomers to the growing fibril becomes thermodynamically favored [210].

It would almost be blasphemous not to be mentioning Finke and Watzky’s Ockham Razor model [211]. Originally intended for modelling kinetics of metal nanoclusters, this model gained a surprising feat in describing amyloid kinetics at a macroscopic level [212]. This model can schematically be represented with two equations: M → M’ (rate constant k) and M + M’ → 2M’ (rate constant k’), where M represents free monomer in solution and M’ represents monomer unit in a forming polymer. The main drawback of the Finke–Watzky and other closely related models, such as the three-step nucleated conformational conversion (NCC) aggregation model [213], and the Gibson–Murphy two step aggregation model, is that they describe the kinetics in an averaged out way with the fine microscopic details remaining largely uncharted [214,215].

The models discussed so far were biased on the fact that nucleation was considered a single time event, treated as a discrete step in the mechanism with elongation necessarily following the nucleation event. However, the forming fibrils can also initiate nucleation on their surfaces in a process termed as secondary nucleation [216,217,218,219]. There even occur heterogenous or cross-seeding events, which account for the heterogeneity observed in neurofibrillary tangles, and closely related amyloid peptide fragments in the aggregates [220,221]. This further sparks risks of formation of hybrid aggregates affecting species, earlier immune to such aggregations, in an inter-species jump [151]. Moreover, the depolymerization event was being considered as the liberation of a single monomer unit from the extending fibril. However, it is plausible that a chunk of the formed fibril dissociates from the parent fibril in a fragmentation process [222]. In a reverse process, the fibril elongation in turn may be aided by coagulation of the fibril chunks. Coagulation is an important elongation process, perhaps not in the initial stages of fibril formation but in the later stages when the free monomer concentration is appreciably depleted. Coagulation may also assist in formation of the seeding nuclei at various stages [222,223]. As discussed earlier in the protein aggregation mechanisms, free monomers in solution and the monomers incorporated into the fibril may have a stark conformational difference. As such, the monomers may first associate with the elongating fibril, forming an intermediate species before being incorporated into the fibril in the required conformation, in a process called locking and docking [224]. In the initial days of amyloid chemistry, it was believed that the fibrillar topology was necessarily unbranched and was backed up by EM and AFM studies of the same. However, the subsequent development in modeling prowess yielded solutions which showed that there should in fact be no major energy barrier for lateral association [225]. Secondary nucleation on a preformed fibril may lead to lateral association, however the lateral growth may fragment up from the parent fibril to form a separate fibril growth altogether. In recent times, small fibrillar lateral growths have been observed in Aβ fibrils [226]. Smaller aggregates found in close vicinity of the large fibrillar masses further strengthen the lateral association hypothesis [21]. It is important to note that not all the aggregating intermediates lead to ordered fibril formation. The on pathway describes the obligate processes that lead to fibril assembly and off-pathways describe those aggregation intermediates which directly cannot lead to fibril formation [227]. These off-pathway aggregates are reportedly shown to have enhanced toxic effects in vivo [228]. The off-pathway aggregation also contributes to fibril assembly, albeit indirectly. Some recent studies, however, discourage the practice of classifying oligomers unstructured precursors to the amyloid fibrils on a binary basis, either on or off-pathway, and stress on a broader and more encompassing definition. 

**Figure 8:**
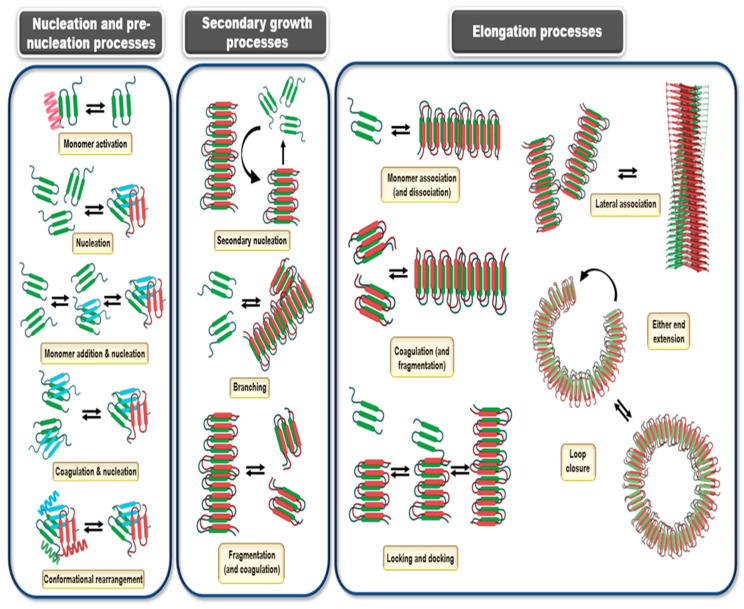
Self-assembly of amyloid fibrils is triggered and facilitated by a number of preceding processes, which include prenucleation and nucleation, secondary growth, and elongation processes, occurring both chronologically and simultaneously to yield the thermodynamically stable fibrils.

**Figure 9:**
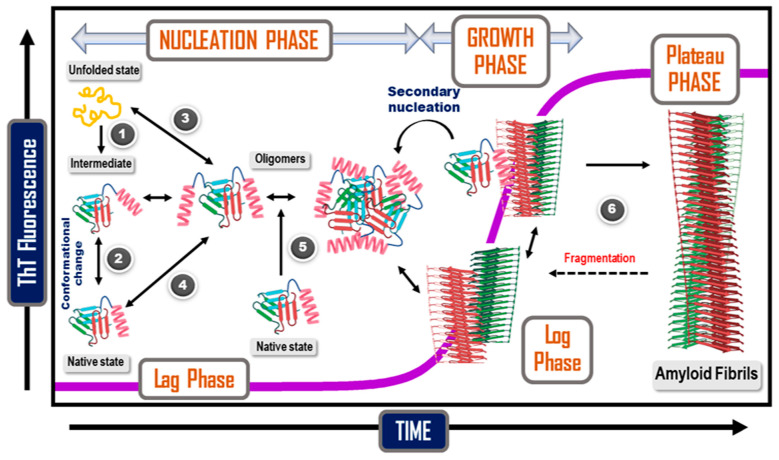
Sigmoidal kinetic curve for amyloid formation probed by using ThT fluorescence. Thioflavin T bound to polymeric amyloid fibrils and increase in fluorescence intensity signaled increased formation of amyloid fibrils. The nascent unfolded polypeptide may either drop down into a native folding cascade via partially folded intermediates (1) and/or may directly be recruited in nucleus formation (3). The native monomer similarly can either undergo partial unfolding (2) and/or associate with the forming nucleus (4,5). When concentration of amyloid fibrils increases significantly, the secondary processes (6) take over as the dominant.

The morphology of amyloid fibrils in nature can be even more bizarre. Apart from the fibrillar species we discussed so far, closed circular loop fibrils are also found along with the regular fibrillar aggregates in protein systems, such as HSA, FtsZ (from *E. coli*), α-synuclein, β-lactoglobulin, α_S2_-casein, crystallin proteins, and apoC-II [229,230,231,232,233,234,235,236] (Figure 8). These closed-loop fragments negatively regulate fibrillation. The incompletely formed fibrils undergo an end-to-end circular association, thus forming a thermodynamic sink for elongating fibrils [237]. The kinetic models for amyloid self-assembly were developed mainly for fitting into empirical curves rather than for prediction purposes. As we mentioned earlier, almost all of these kinetic models bear the same essence: a sigmoidal growth curve described with a lag phase, an exponential growth phase, and a plateau phase (Figure 9). The different flavors added cumulatively closely fit to the empirical data and deconvoluted the underlying microscopic processes. For instance, by considering secondary nucleation and associated secondary processes, including appropriate terms into the rate law, a closer and more realistic fit to the kinetic data can be obtained. The integrated rate law utilizing such an approach was postulated by Cohen et al. [238,239,240].

$$\frac{M\left(t\right)}{M\left(\infty \right)}=1-{\left(\frac{B_{+}+C_{+}}{B_{+}+C_{+}e^{\kappa t}}\frac{B_{-}+C_{+}e^{\kappa t}}{B_{+}+C_{+}}\right)}^{\frac{k_{\infty }^{2}}{\kappa {\tilde{k}}_{\infty }}}e^{-k_{\infty }t}$$

For a description of the variables and parameters used please refer to Table 1.

The mathematical details of the treatment and the various techniques, such as numerical integration, along with the analytical treatments have been reported and reviewed elsewhere [241,242]. Indeed, it has been found for Aβ_42_ that in the absence or low concentration of fibrils, fibril elongation takes place majorly through primary nucleation processes. However, once a critical fibril concentration is achieved, secondary nucleation takes over as the dominant elongating phenomenon [216,243]. The self-assembly is further promoted by a positive feedback loop existing between the monomeric and fibrillar forms [240,244].

The native monomer similarly can either undergo partial unfolding and/or associate with the forming nucleus [181,183,184]. When the concentration of amyloid fibrils increases significantly, the secondary processes take over as the dominant phenomenon [185].

Analytical approaches to explain the kinetic curve reveal an interesting underlying fact. The lag phase and the exponential phases might not necessarily be corresponding to the nucleation and elongation events. The phases might rather correspond to a combination of different molecular level events. For instance, fibril elongation by primary processes might correspond to the slower lag phase, while secondary nucleation and fragmentation to form additional nuclei might be the major contributors in the rapid exponential growth phase [243].

## 13. The Clinical Effects of Amyloid Formation

Amyloidosis refers to various disorders that result from a protein misfolding in which soluble proteins aggregate and convert to insoluble amyloid fibrils [242]. As a result, functional and structural organ damage can be caused by these amyloid deposits [47,245,246].

The extracellular deposition of amyloid fibrils in tissues are involved in different human diseases. Examples include Alzheimer’s disease, Parkinson’s disease, dialysis-related amyloidosis, type-II diabetes, etc. (Table 2) [246]. In amyloidosis, amyloid fibrils build up in tissues, leading to various symptoms, such as reduction in weight, loss in energy, spleen augmentation, tongue augmentation, weakness, bleeding, swelling of legs, numbness, etc. [247,248]. Amyloid depositions can sometimes be limited to one organ or site of the body, known as localized amyloidosis. In contrast, the fibril deposition found in various organs and tissues in the body is termed as systemic. An example of localized amyloidosis is found in Alzheimer’s disease and diabetes mellitus [248]. Localized amyloid can be found in skin, eyelid, conjunctiva, breast, larynx, bronchial tree, lung, and genitourinary tract. In general, low counts of clonal plasma cells can be detected in the biopsy sample [249]. In systemic amyloidosis, symptoms appear as a result of progressive disease in organs and tissues, such as in light chain (AL) amyloidosis and transthyretin amyloidosis (ATTR) [250]. 

Alzheimer’s disease (AD) is considered a complicated neurodegenerative disorder. The main histopathological characteristics of AD are plaques and neurofibrillary tangles that Alois Alzheimer previously described [251]. It has been believed that the building up of amyloid β-peptide (Aβ) in neural tissue is one of the main causes of AD pathogenesis. After the accumulation of (Aβ), the production of neurofibrillary tangles consisting of tau protein results as a consequence of imbalance between the production and clearance of Aβ species [246]. There are various indications about the amyloid hypothesis that regard amyloids as the toxic cause of neural/synaptic damage and dementia. For example, the existence of amyloid in neuritic plaques in AD, the genetics of inherited AD, the contribution of mutations of amyloid precursor protein (APP), Alzheimer-like changes that are found in middle-aged patients with Down syndrome, the molecular biology of Aβ production from APP, the neurotoxicity of amyloid in tissue culture, and detection on transgenic mouse models of AD with human mutant genes, are all indicators of the amyloid hypothesis of AD. It has been demonstrated that AD, known by plaques Aβ peptide, and neurofibrillary tangles (NFTs), composed of intracellular filaments aggregation of hyperphosphorylated tau protein [252]. According to the amyloid cascade hypothesis, the deposition of Aβ induces the pathology of tau protein via induction of the intraneuronal production of NFTs which consist of hyperphosphorylated tau protein [253,254]. Therefore, there has been enormous research demonstrating the association between Aβ and AD. Moreover, there are abundant therapeutic approaches being developed to reduce the production and accumulation of Aβ in the brain that might lead to preventing AD [255,256]. On the other hand, it has been indicated that there is no correlation between the number of amyloid deposits in the brain and the degree of cognitive impairment that the patient has. For example, there are humans without symptoms of AD, but they have Aβ deposits [257].

Neuritic plaques consisting of the high level of insoluble Aβ in the brain parenchyma is a pathology often associated with AD. In addition, the neurofibrillary tangles in various conditions might have the same significant morphological characteristics, but could have a unique structure of tau isoforms [258]. There is no agreement over the stage classification of AD between clinicians, neuropsychologists, and pathologists. Research efforts on disease mechanisms are largely hindered by the lack of an in-life diagnostic test [166]. It has been demonstrated that immunization of AD patients with full-length Aβ_42_ (AN-1792) removed amyloid plaques from the brain. However, it could not stop the neurodegenerative progression [259,260]. The mechanism that causes progress in neurodegeneration is not clear. Excess buildup of misfolded hyperphosphorylated tau causing more neuron damage might be the closest hypothesis to explain it. An additional reasonable rationale might be that a specific construction or constructions of Aβ cause the death of several neurons, a typical of the condition. There is an indication that both the solubility and the amount of Aβ in various pools could be highly relevant in AD. Imaging technologies, utilizing amyloid imaging agents depending on the chemical structure of histologic dyes, can be used to investigate amyloid pathology during disease progression in AD patients [256]. Various unsuccessful clinical attempts of anti-Aβ drugs have been made so far. In contrast, in September 2016, a study revealed that an anti-amyloid antibody diminished Aβ levels in the brain and reduced the rate of failure of cognitive action in people with medium or preclinical Alzheimer’s condition [261]. However, not all researchers are persuaded that Aβ leads to Alzheimer’s and are introducing a diversity of other reasonable inhibitors for the harmful array of signaling cascades that eventually drowns brain cells in AD [16]. The US FDA approved aducanumab, a human IgG1 (the most abundant immunoglobulins in the human serum) anti-Aβ monoclonal antibody selective against Aβ aggregates as the most important disease-modeling cure for Alzheimer’s [262]. Lecanemb was raised as an anti-Aβ protofibril antibody. The drug successfully reduces the aggregated Aβ in the brain. Therefore, it is approved by FDA [263].

Amyloid precursor protein (APP) and Aβ species, which control neurodegeneration, develop via the activation of the protein kinase signaling cascade in AD. This highlights the influential role of protein kinase that should be utilized in the treatment of AD patients [264]. Recently, an interesting study was conducted with mice carrying human Aβ only in their hepatic cells. The study indicated that the protein was migrated through blood via lipoproteins containing a high amount of triglyceride, like it is in humans, and finally diffused into the brain. The mice displayed considerable neurodegeneration and brain atrophy, which was related to neurovascular inflammation and dysfunction of cerebral capillaries, both generally noticed in AD. This indicates that there is a possibility of induction of neurodegeneration by peripherally derived Aβ species, suggesting that Aβ produced in the liver might be an important factor in human AD [265]. Based on the amyloid cascade hypothesis, Aβ is produced by the sequential cleavage of the transmembrane protein amyloid precursor protein (APP) via α,β and γ secretases. The cleavage of APP at the plasma membrane is controlled by α-secretase, while β and γ secretases govern the production of Aβ via amyloidogenic pathway [246,263]. Therefore, manipulating the β and γ secretases cloud reduces the production of Aβ and prevent its accumulation from depositing into soluble oligomers and fibrils that initiate oxidative injury, microglial and astrocytic activity besides prevention kinase/phosphatase activity that cause neuronal death [266]. Furthermore, elevation and aggregation of Aβ are correlated with the chronic response of the innate immune system by activating microglia via various immunological receptors [267]. Despite the various trials of anti-Aβ therapies, there is evidence that pathogens trigger soluble Aβ to aggregate into insoluble deposits to prevent the leaking of the pathogen or stop it [268]. For example, recently, it has been indicated that there is a correlation between Aβ and protection and repair of the central nervous system due to the antimicrobial and antiviral function of Aβ oligomer and consecutive fibril formation [269].

On the other hand, some researchers believe that tau protein is more relevant in dementia, and consequently suggest targeting tau protein may be a more meaningful approach than focusing on the development of anti-amyloid therapy in protecting corporeal action in Alzheimer’s disease. However, it should be noted that improvement the effectiveness of the therapy with tau protein is not an easy task. There are various antibodies for the protein, and two vaccines are currently under clinical investigation. As a treatment for AD, tau protein is still in its nascent stage [270]. In AD, amyloid deposits accumulate to produce plaques in the extracellular space, and tau proteins occur in the form of neurofibrillary tangles present in the intracellular spaces that are important hallmarks in the final post-mortem of clinical AD cases [271,272]. It is believed that Alzheimer’s disease typically begins at an age of 15–20 years in people with a family history of AD and 20–30 years in sporadic people before indication of any clinical symptoms [273,274]. Unfortunately, when the clinical phenotype is identified, significant neuronal and synaptic degeneration and massive neuroinflammatory changes have already occurred, ushering the need for early detection of AD [270]. 

## 14. Prion

Prion diseases are infectious neurodegenerative diseases that are almost always fatal [33]. Prion diseases are characterized by the accumulation of the protease-resistant prion protein in the central nervous system, which sometimes forms amyloid-like plaques [275]. Although the exact mechanism is not known, it is believed that the conversion of the cell-surface glycoprotein (PrPC) to conformationally altered isoform (PrPSc) when the infectious materials of PrPSc interact with the host PrPC causes the replication of prion [33,276]. The conformational change of the human prion protein into a self-replicating amyloid-like form is associated with a class of fatal diseases, transmissible spongiform encephalopathies [276,277]. When caused by spontaneous prion misfolding, they lead to sporadic Creutzfeldt–Jakob disease, while mutations in the prion protein gene cause familial Creutzfeldt–Jakob disease, Gerstmann–Sträussler Scheinker syndrome, and fatal familial insomnia [276,278]. In this context, both prion and Aβ and prion protein are related to neurodegenerative disorders, such as Creutzfeldt–Jakob and Alzheimer’s, respectively. However, prions are related to neurodegenerative diseases that occur in humans and animals [279]. It was considered that prions are unique because they are infectious. However, it has been indicated that that Aβ proliferates in a manner that prions do [280].

## 15. Future Perspectives

Proteins assemble and convert to insoluble aggregates that cannot be degraded and amyloid fibrils, related to various diseases, are produced. Some of the frequent amyloid diseases are Alzheimer’s disease, diabetes type-II, and mad cow disease [47].

**Figure 10:**
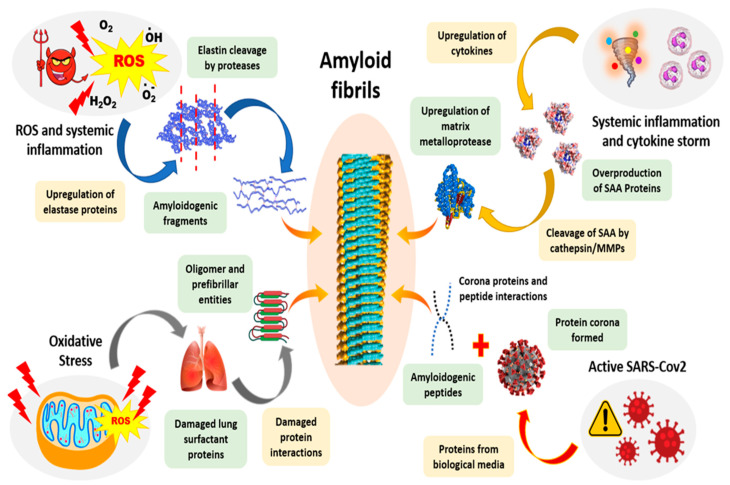
SARS-CoV-2 induced acute respiratory syndromes lead to upregulation of ROS and inflammation that can potentially lead to amyloidosis.

Sever acute respiratory syndrome corona virus 2 (SARS-CoV-2), the causative of the coronavirus-19 (COVID-19 ) pandemic, is a virus that needs no introduction in contemporary times at least. Intriguingly, there have been recent hypotheses and evidence suggesting a possible link between COVID-19 and amyloidosis [281] (Figure 10). The first observation of amyloidosis in such cases of acute respiratory inflictions dates back to 2002 wherein the patients showed a buildup of elastin amyloids. Such respiratory syndromes and viral infections are almost ubiquitously involved with cytokine storm, which upregulates metalloprotease expressions [282,283,284]. These in turn cleave the serum amyloid A proteins, generating a pool of potential amyloidogenic peptides [285]. Furthermore, these cases are also possibly responsible for the upregulation of elastase enzymes, which free elastin fibers from their intrinsic mesh and coerce them into amyloidogenic aggregations [286]. AA amyloidosis is hypothesized to be responsible for renal, lung and multiple organ failures in COVID-19 [287]. ARDS cases are associated with extreme conditions of oxidative stress. Oxidative stress can both directly and indirectly lead to amyloidosis. The leakage of reactive oxygen species from mitochondria is possibly enhanced, leading to more frequent misfolding in proteins, possibly driving amyloidosis [287]. Another hypothesis in place suspects that these viral infections might bring about biochemical changes at cellular levels, favoring the release of amyloidogenic peptides and eventually leading to amyloidosis [288,289].

Heme-Aβ has been shown to be involved in the release of partially reduced oxygen species (PROS) during the TCA cycle in mitochondria [289,290]. These PROS are mainly responsible for the degradation of neurotransmitters and have been shown to be associated with Alzheimer’s disease. The correlation of increased oxidative stress with SARS-CoV-2 infection further sparks possibilities of early AD onset in COVID-19 affected patients.

More recently, synthetic amyloids have also gained clout as important functional next-generation biomaterials, finding uses in drug delivery, food industry, vaccines, water purification, tissue engineering, and as biosensors [291,292,293,294,295,296].

Recently, Biogen and Eisai’s drug Aducanumab, a monoclonal antibody against β-amyloid was approved by the FDA. ADUHELM™ is the first-ever and the only drug as of yet that addresses the apparent pathology of Alzheimer’s disease [297]. The root cause of AD remains debated with β-amyloid aggregation in the AD brain, being mostly completed in the early clinical stages, while tau protein continues to accumulate throughout the course of the disease [166,298]. Accordingly, amyloids have emerged as topic of interest in physical, chemical, biological, and medical research. Advancement in biophysical techniques, such as solid-state nuclear magnetic resonance spectroscopy (ssNMR), X-ray and electron diffraction cryo-electron microscopy (cryo-EM), and mass spectrometry, which assist in analysis of amyloids, aim to provide a better understanding of the molecular mechanisms underlying the amyloid cascade. It is an exhilarating time to live in indeed, to see what the future holds for the cure of AD and amyloid research.

## Figures and Tables

**Table 1:** Variable description for Amyloid Kinetics Rate Law by Cohen et al. [227,228,229].

Variable	Description
M(t)	Total fibril mass at a time t
M(∞)	Final fibril mass
$k_{n}$	Rate constant for primary nucleation
$k_{+}$	Rate constant for monomer addition and elongation
$k_{off}$	Rate constant for depolymerization
$k_{-}$	Rate constant for fragmentation
$k_{2}$	Rate constant for secondary nucleation
m(0)	Initial concentration of soluble monomers in solution
$n_{c}$	Rate order with respect to monomer (primary pathway)
$n_{2}$	Rate order with respect to monomer (secondary pathway)
λ	$\sqrt{2k_{+}k_{n}m{\left(0\right)}^{n_{c}}}$
κ	$\sqrt{2k_{+}k_{2}m{\left(0\right)}^{n_{2}+1}}$
$B_{\pm }$	$\frac{k_{\infty }\pm {\tilde{k}}_{\infty }}{2\kappa }$
$C_{\pm }$	$\pm \frac{\lambda^{2}}{2\kappa^{2}}$
$k_{\infty }$	$\sqrt{\frac{2\kappa^{2}}{n_{2}\left(n_{2}+1\right)+\frac{2\lambda^{2}}{n_{c}}}}$
${\tilde{k}}_{\infty }$	$\sqrt{k_{\infty }^{2}-4C_{+}C_{-}\kappa^{2}}$

**Table 2:** Various amyloid related diseases.

S.No.	Disease	Protein/Peptide	Structure
1.	AD (Alzheimer’s Disease)	Aβ (β-Amyloid peptide)	Natively unfolded
2.	HCHWA (Dutch Hereditary Cerebral Hemorrhage With Amyloidosis)	Aβ (β-Amyloid peptide)	Natively unfolded
3.	(CAA) Cerebral Amyloid Angiopathy	Aβ (β-Amyloid peptide)	Natively unfolded
4.	Familial British dementia	Cystine Cross-linked Amyloid Bri	Natively unfolded
5.	(HD) Huntington Disease	Huntingtin	Unfolded exon 1 which forms fibrils
6.	SBMA (Spinal and Bulbar DNA-binding Muscular Atrophy)	Androgen receptor protein	Ligand-binding and domains α-helical; N-terminal—natively unfolded
7.	NIID (Neuronal Intranuclear Inclusion Disease)	Ataxin-1	Natively unfolded
8.	SCA (Spinocerebellar Ataxia)	Ataxin-1	Natively unfolded
9.	DRPLA (hereditary Dentatorubral Pallidoluysian Atrophy)	Atrophin-1	Probably natively unfolded
10.	Type II Diabetes Mellitus	Amylin	Natively unfolded
11.	MCT (Medullary Carcinoma of Thyroid)	Calcitonin	Natively unfolded
12.	PD (Parkinson’s disease)	α-Synuclein	Natively unfolded
13.	DLBD (Diffuse Lewy Bodies Disease)	α-Synuclein	Natively unfolded
14.	LBVAD (Lewy Bodies Variant of Alzheimer’s Disease)	α-Synuclein	Natively unfolded
15.	DLB (Dementia with Lewy Bodies)	α-Synuclein	Natively unfolded
16.	MSA (Multiple System Atrophy)	α-Synuclein	Natively unfolded
17.	Pick’s Disease	Tau protein	Natively unfolded
18.	PSP (Progressive Supranuclear Palsy)	Tau protein	Natively unfolded
19.	CJD (Creutzfeldt–Jakob Disease)	Prion protein	N-terminal fragment— natively unfolded; C-terminal domain: α-helical
20.	GSS (Gerstmann–Straussler Schneiker Syndrome)	Prion protein	N-terminal fragment— natively unfolded; C-terminal domain: α-helical
21.	FII (Fatal Familial Insomnia)	Prion protein	N-terminal fragment— natively unfolded; C-terminal domain: α-helical
22.	Kuru	Prion protein	N-terminal fragment— natively unfolded; C-terminal domain: α-helical
23.	BSE (Bovine Spongiform)	Prion protein	N-terminal fragment— natively unfolded; C-terminal domain: α-helical
24.	Scrapie	Prion protein	N-terminal fragment— natively unfolded; C-terminal domain: α-helical
25.	Spongiform Encephalopathy	Prion protein	N-terminal fragment— natively unfolded; C-terminal domain: α-helical
26.	Amyotrophic lateral sclerosis	Superoxide dismutase 1	β-sheet and Ig-like
27.	Familial Amyloidotic Polyneuropathy	Transthyretin mutants	β-sheet
28.	Amyloid light chain (AL) amyloidosis	Immunoglobulin (Ig) light chains	β-sheet and Ig-like
29.	Senile systemic amyloidosis	Wild-type transthyretin	β-sheet
30.	Haemodialysis-related amyloidosis	β2-microglobulin	β-sheet and Ig-like
31.	Lysozyme amyloidosis	Lysozyme mutants	α-helical and β-sheet
32.	Apolipoprotein A1 amyloidosis	Apo A-1 fragments	Intrinsically disordered
33.	Injection-localized amyloidosis	Insulin	α-helical and insulin-like
34.	Progressive supranuclear palsy	Tau protein	Intrinsically disordered
35.	Argyrophilic grain disease	Tau protein	Intrinsically disordered
36.	Tangle predominant dementia	Tau protein	Intrinsically disordered
37.	Chronic traumatic encephalopathy	Tau protein	Intrinsically disordered
38.	Ganglioglioma	Tau protein	Intrinsically disordered
39.	Meningioangiomatosis	Tau protein	Intrinsically disordered
40.	Subacute sclerosing panencephalitis	Tau protein	Intrinsically disordered
41.	Lead encephalopathy	Tau protein	Intrinsically disordered
42.	Tuberous sclerosis	Tau protein	Intrinsically disordered
43.	Hallervorden–Spatz disease	Tau protein	Intrinsically disordered
44.	Lipofuscinosis	Tau protein	Intrinsically disordered
45.	Familial Danish dementia	Tau protein	Intrinsically disordered
46.	Heavy-chain amyloidosis	Fragments of immunoglobulin heavy chain	All-β, Ig-like
47.	AA amyloidosis	Full or N-terminal fragments of serum amyloid A protein (SAA)	All-α, SA-like four-helix bundle
48.	Hereditary visceral amyloidosis	β_2_-microglobulin	All-β, Ig-like
49.	ApoAII amyloidosis (mainly renal)	C-term extended apolipoprotein A-II	Unknown
50.	ApoAIV amyloidosis (many organs)	N-term fragments of apolipoprotein A-IV (ApoAIV)	Unknown
51.	ApoCII amyloidosis	Apolipoprotein C-II	All-α, unknown fold
52.	Fibrinogen amyloidosis	Fragments of fibrinogenα-chain	Unknown
53.	Atrial amyloidosis	Atrial natriuretic factor(ANF)	Intrinsically disordered
54.	Pituitary prolactinoma	N-term fragments of prolactin (PRL)	Unknown
55.	Aortic medial amyloidosis	Medin	Intrinsically disordered
56.	Gelatinous drop-like corneal dystrophy	Lactotransferrin	α + β, periplasmic binding protein-like II
57.	Calcifying epithelial odontogenic tumors	Odontogenic Ameloblast Associated protein (ODAM)	Unknown
58.	Pulmonary alveolar proteinosis	Pulmonary surfactant-associated protein C (SP-C)	All α-transmembrane helical fragment
59.	Renal amyloidosis	Leukocyte cell-derived chemotaxin-2 (LECT-2)	Unknown
60.	Lattice corneal dystrophy, type 1	C-term fragments of kerato-epithelin	Unknown
61.	Lattice corneal dystrophy, type 3A	C-term fragments of kerato-epithelin	Unknown
62.	Lattice corneal dystrophy, Avellino type	C-term fragments of kerato-epithelin	Unknown
63.	Seminal vesicle amyloidosis	Semenogelin-1 (SGI)	Unknown
64.	Prostate cancer	All-α, EF hand-like	Proteins S100A8/A9
65.	Injection-localized amyloidosis	Enfuvirtide	Unknown
66.	Frontotemporal dementia with Parkinsonism	Tau protein	Natively unfolded
67.	Icelandic hereditary cerebral amyloid angiopathy	Mutant of cystatin C	A + β, cystatin-like
68.	Spinocerebellar ataxia 17	TATA box-binding protein with polyQ expansion	A + β, TBP-like
69.	Hereditary cerebral haemorrhage with amyloidosis	Mutants of amyloid β	Natively unfolded
70.	Cataract	γ-Crystallins	All-β, γ-crystallin-like
71.	Pulmonary alveolar proteinosis	Lung surfactant protein C	Unknown
72.	Inclusion-body myositis	Amyloid β peptide	Natively unfolded
73.	Cutaneous lichen amyloidosis	Keratins	Unknown
74.	Secondary systemic amyloidosis	Serum amyloid A	Unknown
75.	Familial Amyloid AApoA1	Polyneuropathy II	Unknown
76.	Hereditary nonneuropathic systemic amyloidosis	ALys	Unknown

## Abbreviations

AD: Alzheimer’s; PKU: Phenylketonuria; ThT: Thioflavin T; CR: Congo Red; APP: Amyloid Precursor Protein; CuBD; Copper-Binding Domain; GFLD: Growth Factor-Like Domain; AICD: APP intracellular C-terminus domain; CTF: C-terminal fragment; IAPP: Islet amyloid polypeptide; BACE: Beta-site APP Cleaving Enzyme; CD: circular dichroism spectroscopy; TEM: transmission electron micriecopy;FGF-1: newt acidic fibroblast growth factor; TRR: transthyretin; TFE: 2,2,2-trifluoroethanol;ANS:1-anilinonaphthalene8-sulfonate; BIS-ANS:4,4′-bis-1-anilinonaphthalene 8-sulfonate; FTIR: Fourier Transform Infrared; AFM: atomic force microscopy; ssNMR: sloid state nuclear magnetic resonance spectroscopy; HPLC: high pressure liquid chromatography; ATTR: Transthyretin amyloidosis; CTF: Carboxy terminal fragment; NEP: Neprilysin; ECE: Endothelin Converting Enzyme; MMP: Matrix Metalloprotease; IDE: Insulin Degrading Enzyme; LDLP: Low Density Lipoprotein; LRPs: Low Density Lipoprotein receptor related proteins; RAGE: Receptor for Advanced Glycation End products; SDS: Sodium Dodecyl Sulfate; NHPs: Non-Human Primates; CBAs: Competing Basins of Attractions; NBAs: Native Basins of Attraction; PES: Potential Energy Surface; IL: Interleukin; ILRA: Interleukin Receptor antagonist; IFN: Interferon; G-CSF: Granulocyte Colony Stimulating Factor; NDP: Nucleation Dependent Polymerization; NCC: Nucleated Conformational Conversion; SARS-CoV-2: Severe Acute Respiratory Syndrome Coronavirus 2; COVID-19: Coronavirus Disease 2019.
